# Rhes Counteracts Dopamine Neuron Degeneration and Neuroinflammation Depending on Gender and Age

**DOI:** 10.3389/fnagi.2018.00163

**Published:** 2018-05-31

**Authors:** Giulia Costa, Annalisa Pinna, Pier Francesca Porceddu, Maria Antonietta Casu, Anna Di Maio, Francesco Napolitano, Alessandro Usiello, Micaela Morelli

**Affiliations:** ^1^Department of Biomedical Sciences, Section of Neuropsychopharmacology, University of Cagliari, Cagliari, Italy; ^2^National Research Council of Italy, Neuroscience Institute, Cagliari, Italy; ^3^National Research Council of Italy, Institute of Translational Pharmacology, UOS of Cagliari, Scientific and Technological Park of Sardinia POLARIS, Pula, Italy; ^4^IRCCS Casa Sollievo della Sofferenza, San Giovanni Rotondo, Italy; ^5^Laboratory of Behavioral Neuroscience, Ceinge Biotecnologie Avanzate, Naples, Italy; ^6^Department of Molecular Medicine and Medical Biotechnology, University of Naples “Federico II”, Naples, Italy; ^7^Department of Environmental, Biological and Pharmaceutical Sciences and Technologies, University of Campania, Caserta, Italy; ^8^National Institute of Neuroscience (INN), University of Cagliari, Cagliari, Italy

**Keywords:** microglia, astrocytes, dopamine, Parkinson’s disease, caudate putamen, substantia nigra pars compacta

## Abstract

We have recently shown that male Rhes knockout (KO) mice develop a mild form of spontaneous Parkinson’s disease (PD)-like phenotype, characterized by motor impairment and a decrease in nigrostriatal dopamine (DA) neurons. Experimental evidence has implicated neuroinflammation in PD progression, and the presence of activated glial cells has been correlated with DA neuron degeneration. Despite this, several factors, such as gender, have been found to affect DAergic neuron degeneration and influence neuroinflammation, explaining the differences between men and women in the etiology of PD. On these basis, we studied age and gender differences in DA neuron degeneration and gliosis in the nigrostriatal system of adult (3-month-old) and middle aged (12-month-old) male and female Rhes wild-type (WT) and KO mice. Through immunohistochemistry, tyrosine hydroxylase (TH), microglial (complement type 3 receptor [CD11b]) and astroglial (glial fibrillary acid protein [GFAP]) increase, were evaluated. Adult male Rhes KO mice showed a decrease in TH and an increase in CD11b, both in the caudate putamen (CPu) and substantia nigra pars compacta (SNc), and an increase in GFAP in the CPu. In contrast, adult female Rhes KO mice showed only a decrease in TH in the SNc, whereas no modifications to the levels of GFAP and CD11b were observed in the CPu or SNc. Middle aged male Rhes KO mice showed a decrease in TH in the CPu and SNc, and an increase in GFAP and CD11b in the SNc. Middle aged female Rhes KO mice showed a decrease in TH in the CPu and SNc and an increase in CD11b only in the CPu, but no modifications to GFAP levels. The more marked DA neuron degeneration and neuroinflammation in male compared with female Rhes KO mice, while confirming the role of Rhes as an important protein for DA neuron survival, gives support to Rhes KO mice as a valuable preclinical model for studying the vulnerability factors of DA neuron degeneration as in PD.

## Introduction

Parkinson’s disease (PD) is a chronic neurodegenerative disorder characterized by the degeneration of dopaminergic (DAergic) neurons in the substantia nigra pars compacta (SNc; Fuzzati-Armentero et al., 2015). The mechanisms responsible for the degeneration observed in PD have remained controversial, but several factors, such as neuroinflammation, oxidative stress, excitotoxicity, reduced expression of trophic factors, and dysfunction of the protein degradation system, may participate in the pathogenesis of nigrostriatal neuron death (Greenamyre and Hastings, 2004; Schapira and Jenner, 2011). Although several genes that cause certain forms of inherited PD have been identified, most of them are still not characterized, or require further study aimed at clarifying the interplay between the gene and other possible pathogenic factors (Klein and Westenberger, 2012).

Rhes is a small guanosine triphosphate-binding protein that is highly expressed in the caudate putamen (CPu; Falk et al., 1999; Vargiu et al., 2004). Rhes mRNA is localized in striatal GABAergic medium-sized projection neurons of rodents and humans (Errico et al., 2008; Ghiglieri et al., 2015; Vitucci et al., 2015) and in large aspiny cholinergic interneurons (Sciamanna et al., 2015), where it modulates dopamine (DA)-dependent transmission. Recently, we have shown that Rhes mRNA is also expressed, although to a lesser extent, in tyrosine hydroxylase (TH)-positive neurons of the SNc and ventral tegmental area of the mouse midbrain (Pinna et al., 2016). Consistent with Rhes mRNA midbrain localization and its putative protective role in DAergic cell survival, male Rhes knockout (KO) mice have shown a reduction in TH-positive neurons in the SNc, associated with a progressive deficit in motor coordination and balance (Pinna et al., 2016).

Among the possible pathogenic mechanisms involved in PD initiation/progression proposed above, emerging evidence indicates that a chronic neuroinflammatory reaction, driven mainly by reactive microglia, may play a vital pivotal role in the process of neuronal loss (Barcia et al., 2009; Tansey and Goldberg, 2010; Halliday and Stevens, 2011; Hirsch et al., 2012; Phani et al., 2012). The first evidence of increased microglial activation in post-mortem brains of PD patients was published by McGeer et al. (1988) and, since then, microgliosis has been thought to play a role in PD. Although microglia are the primary activated cells, under pathological conditions, astroglial cells may contribute to the neuroinflammatory response, which leads to the DA neuron degeneration observed in PD (Teismann and Schulz, 2004; Maragakis and Rothstein, 2006). In line with this, several studies have reported an increase in the number of astrocytes, identified with the glial fibrillary acidic protein (GFAP), in experimental models of PD (Ciesielska et al., 2009; Costa et al., 2013, 2014).

On the other hand, clinical and experimental studies indicated that gender might constitute a factor of vulnerability for both neuron degeneration and glial cell activation. One of the factors protecting from PD are estrogens, and in particular estradiol (Gillies et al., 2014), an issue particularly important in relation to aging (Dhandapani and Brann, 2002). Coinciding with the estrogen deficit that occurs at menopause, the risk of PD increases dramatically (Labandeira-Garcia et al., 2016). Nevertheless, epidemiological studies have revealed that, after aging, male gender is a prominent risk factor for development of PD (Gillies et al., 2014). Large meta-analysis studies revealed that men are approximately twice as likely as women to develop the disease, although some studies reported male to female ratios up to 3.7 (Labandeira-Garcia et al., 2016). Thus, it is reasonable to speculate that both age and gender may influence the nigrostriatal neuron degeneration previously observed in male Rhes KO mice.

Considering the influence of Rhes on the survival of nigrostriatal DAergic neurons (Pinna et al., 2016), and the differential incidence of PD between men and women (Gillies et al., 2014; Labandeira-Garcia et al., 2016), in the present study, we investigated the possible presence of neurotoxic and neuroinflammatory events in male and female Rhes KO mice at different ages. Neurodegeneration was assessed by means of immunoreactivity of TH, the enzyme involved in the synthesis of DA, whereas neuroinflammation was evaluated by measuring microgliosis and astrogliosis through immunoreactivity for complement type 3 receptor (CD11b) and GFAP respectively, in the CPu and SNc.

## Materials and Methods

### Animals and Treatments

Adult (3-month-old) and middle aged (12-month-old) male and female Rhes wild-type (WT) and Rhes KO mice without PGK-Neo cassette (Sciamanna et al., 2015), backcrossed to F11 generation to the C57BL/6J strain, were used in this study. Mice were housed in groups of 4–6 in standard polycarbonate cages with sawdust bedding and maintained on a 12-h-light/dark cycle (lights on at 8:00 am). Food and water were freely available. All experiments were conducted in accordance with the guidelines for animal experimentation of the EU directives (2010/63/EU; L.276; 22/09/2010) and with the guidelines approved by the Ethical Committee of the University of Cagliari. Experiments were designed to minimize animal discomfort to the least extent possible and to reduce the number of animals used.

### Immunohistochemistry

Mice were anesthetized and transcardially perfused with paraformaldehyde (4% in 0.1 M phosphate buffer, pH 7.4). For each immunohistochemical evaluation, three sections of 50 μm from the CPu (A: 1.10 mm; 0.74 mm; 0.38 mm from bregma) and SNc (A: −2.92 mm; −3.28 mm; −3.64 mm from bregma) were cut coronally on a vibratome. All coordinates were relative to bregma, according to the mouse brain atlas of Paxinos and Franklin (2008). Then, sections were incubated overnight at 4°C with the primary antibody (polyclonal rabbit anti-TH, 1:1000, Millipore, Temecula, CA, USA; monoclonal mouse anti-GFAP, 1:400, Sigma-Aldrich, Milan, Italy; monoclonal rat anti-mouse CD11b, 1:1000, Serotec, Oxford, UK). For diaminobenzidine visualization of TH, GFAP, and CD11b, the proper biotinylated secondary antibody (goat anti-rabbit immunoglobulin G (IgG) for TH; goat anti-mouse IgG for GFAP; goat anti-rat IgG for CD11b, all from Vector, Peterborough, UK) was used and the avidin–biotin–peroxidase complex protocol (ABC, Vector, Peterborough, UK) was followed (Costa et al., 2014). Moreover, an additional set of SNc sections was stained with Nissl staining to evaluate cell death in this area.

### Analysis of TH Immunoreactivity in the CPu

Images were digitized (Axio Scope A1, Zeiss, Oberkochen, Germany) in gray scale and captured at 5× magnification. Analysis was performed in a blinded manner in the three sections. The density of immunoreacted fibers was determined quantitatively using the ImageJ program (U.S. National Institutes of Health, USA). No significant differences in the density of immunoreacted fibers were seen between the three sections, thus values from different levels were averaged (Frau et al., 2016).

### Stereological Counting of TH-Immunoreactive Neurons in the SNc

Stereological analysis of the total number and density of TH-positive neurons in the SNc was carried on in a blinded manner on both hemispheres, using a software (Stereologer) linked to a motorized stage on a light microscope (Casu et al., 2004). The SNc region was outlined at low magnification (2×), and sampling of cells was achieved using automatically randomized sampling and an optical dissector (50 × 50 × 15 μm). Cells were sampled with a 40× objective through a defined depth with a guard zone of 2 μm. Coefficient of error ranged from 0.05 to 0.1 (Casu et al., 2004).

### Analysis of GFAP Immunoreactivity

Images were digitized (Axio Scope A1, Zeiss, Oberkochen, Germany) under constant light conditions. Sections were captured at 20× magnification (for CPu analysis) or at 10× magnification (for SNc analysis). Analysis was performed in a blinded manner in the three sections. The number of GFAP (+) cells was determined quantitatively in the dorsolateral and ventromedial CPu, and in the whole SNc, left and right, using the Multi-point tool of ImageJ program. Astroglial cells were counted when a cell body from which processes extended was observed, or when the processes were all directed toward a central point that corresponds with the likely position of the cell body deeper in the tissue. GFAP-expressing fibers without a clear indication of the associated cell body were not counted. For each level of the CPu or SNc, the obtained values from the different levels were averaged (Costa et al., 2014).

### Analysis of CD11b Immunoreactivity

Images were digitized in a gray scale and captured at 10× magnification (for CPu analysis) or at 20× magnification (for SNc analysis). Analysis was performed in a blinded manner in the three sections. The levels of CD11b were determined quantitatively in the dorsolateral and ventromedial CPu, and in the whole SNc, left and right, using the ImageJ program. Before starting the analysis, in the ImageJ dialog box called “Set measurements,” we selected “Mean gray values,” and then the area of interest (SNc or CPu) was selected for each image using the Polygon Selection Tool. In this manner we were able to obtain the average gray value within the selection. This value is the sum of the gray values of all the pixels in the selection divided by the number of pixels. For each level of the SNc or CPu, the obtained values from different levels were averaged (Costa et al., 2014).

### Statistics

Statistical analysis was performed with Statistica for Windows (StatSoft, Tulsa, OK, USA). Data were statistically compared by means of a three-way (gender × genotype × age) analysis of variance (ANOVA). ANOVA analysis was followed by Tukey’s *post hoc* test. Results were considered significant at *p* < 0.05, and the results are expressed as mean ± SEM for every analysis performed.

## Results

### TH Immunoreactivity

Three-way ANOVA analysis of TH immunoreactivity in CPu revealed a significant effect of gender (*F*_(1,48)_ = 30.76, *p* < 0.0001), genotype (*F*_(1,48)_ = 55.64, *p* < 0.0001) and age (*F*_(1,48)_ = 189.19, *p* < 0.0001). Moreover, a significant gender × genotype interaction (*F*_(1,48)_ = 22.90, *p* < 0.0001) and gender × age interaction (*F*_(1,48)_ = 33.21, *p* < 0.0001) were also observed in CPu. Furthermore, three-way ANOVA analysis of the total number of TH-positive neurons in SNc revealed significance of gender (*F*_(1,57)_ = 10.08, *p* < 0.005), genotype (*F*_(1,57)_ = 32.85, *p* < 0.0001) and age (*F*_(1,57)_ = 11.53, *p* < 0.005). Finally, a significant gender × age interaction (*F*_(1,57)_ = 49.79, *p* < 0.0001) was also observed in SNc.

**Figure 1:**
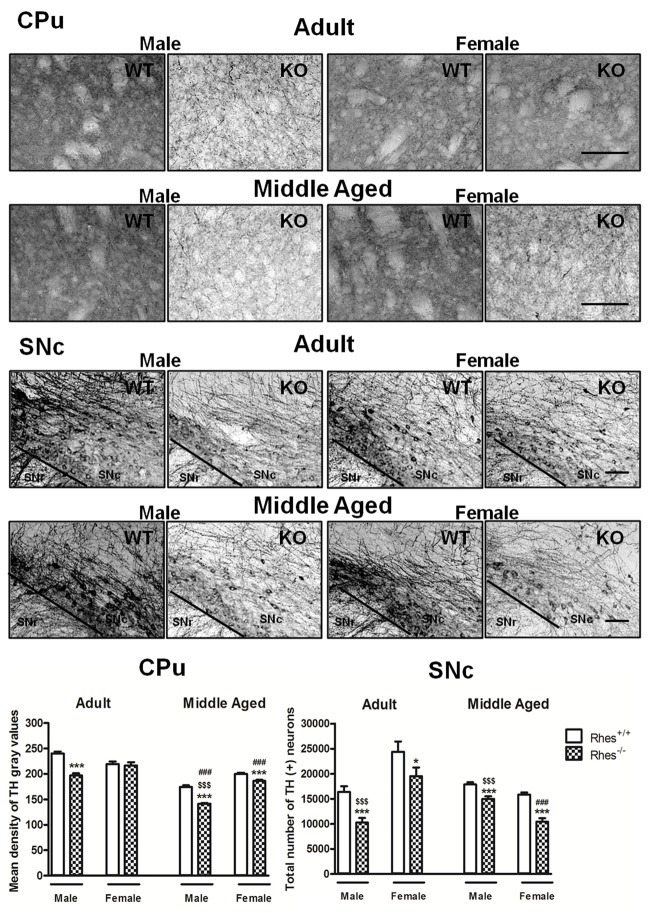
Tyrosine hydroxylase (TH) immunohistochemistry in Rhes wild-type (WT) and Rhes knockout (KO) mice. Representative sections and histograms of the Caudate putamen (CPu) and Substantia nigra pars compacta (SNc) immunostained for TH of adult and middle aged male and female Rhes WT and Rhes KO mice. The histograms in the left panel show the mean density of gray values of TH-positive fibers in the CPu, whereas the histograms in the right panel show the number of TH-positive neurons in the SNc, calculated by stereological analysis. Values are expressed as mean ± SEM. The number of mice per group is: adult Rhes WT, males *n* = 5 and females *n* = 8; middle aged Rhes WT, males *n* = 12 and females *n* = 6; adult Rhes KO, males *n* = 5 and females *n* = 9; middle aged Rhes KO, males *n* = 8 and females *n* = 6. *p < 0.05 and ***p < 0.001 compared with Rhes WT mice; ^$$$^*p* < 0.001 compared with female Rhes KO mice; ^###^*p* < 0.001 compared with the respective adult Rhes KO mice by Tukey’s *post hoc* test. Scale bar: 50 μm.

In particular, adult male Rhes KO mice showed a significant decrease in both the density of TH-positive fibers in the CPu compared with WT mice, and in the total number of TH-positive neurons in the SNc compared with male WT and female Rhes KO mice (Figure 1). In contrast, adult female Rhes KO mice showed a significant decrease in the total number of TH-positive neurons in the SNc compared with female WT mice (Figure 1).

Middle aged male and female Rhes KO mice showed a significant decrease in TH immunostaining, both in the CPu and SNc compared with WT mice (Figure 1). Moreover, middle aged male Rhes KO mice showed a different modification to TH immunostaining in the CPu and SNc compared with middle aged female Rhes KO mice (Figure 1).

Finally, middle aged male Rhes KO mice showed a significant decrease of TH-positive fibers in the CPu compared with the adults, whereas middle aged female Rhes KO mice showed a significant decrease of TH-positive fibers in the CPu and TH-positive neurons in the SNc compared with the adults (Figure 1).

Results obtained with Nissl staining in adult and middle aged Rhes KO mice confirmed the decrease in the total number of TH positive neurons (Supplementary Table S1).

### GFAP Immunoreactivity

Three-way ANOVA analysis of GFAP immunoreactivity in CPu revealed a significant effect of gender (*F*_(1,49)_ = 17.80, *p* < 0.0001), genotype (*F*_(1,49)_ = 4.11, *p* < 0.05) and age (*F*_(1,49)_ = 6.78, *p* < 0.05). Moreover, three-way ANOVA analysis of GFAP immunoreactivity in SNc revealed significance of gender (*F*_(1,49)_ = 5.39, *p* < 0.05), genotype (*F*_(1,49)_ = 5.71, *p* < 0.05) and age (*F*_(1,49)_ = 7.06, *p* < 0.05). Finally, a significant gender × age interaction (*F*_(1,49)_ = 38.52, *p* < 0.0001) was also observed in SNc.

**Figure 2:**
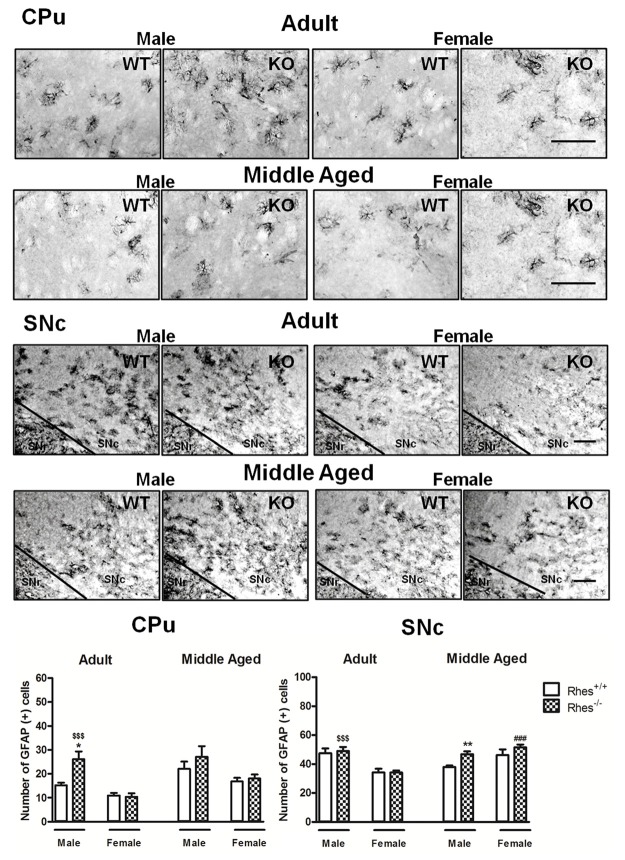
Glial fibrillary acid protein (GFAP) immunohistochemistry in Rhes WT and Rhes KO mice. Representative sections and histograms of the CPu and SNc immunostained for GFAP of adult and middle aged male and female Rhes WT and Rhes KO mice. The histograms in the left and right panels show the number of GFAP-positive cells in CPu and SNc, respectively. Values are expressed as mean ± SEM. The number of mice per group is: adult Rhes WT, males *n* = 5 and females *n* = 8; middle aged Rhes WT, males *n* = 12 and females *n* = 6; adult Rhes KO, males *n* = 5 and females *n* = 9; middle aged Rhes KO, males *n* = 8 and females *n* = 6. *p < 0.05 and **p < 0.005 compared with Rhes WT mice; ^$$$^*p* < 0.001 compared with female Rhes KO mice; ^###^*p* < 0.001 compared with the respective adult Rhes KO mice by Tukey’s *post hoc* test. Scale bar: 50 μm.

In particular, adult male Rhes KO mice showed a significant increase in the number of GFAP-positive cells in the CPu, compared with male WT and female Rhes KO mice (Figure 2). Moreover, an increase in the number of GFAP-positive cells in the SNc was observed compared with female Rhes KO mice (Figure 2). No modifications to the number of GFAP-positive cells were observed in adult female Rhes KO mice, either in the CPu or SNc (Figure 2).

Middle aged male Rhes KO mice showed a significant increase in the number of GFAP-positive cells in the SNc, but not in the CPu, compared with male WT mice, whereas no modifications to the number of GFAP-positive cells were observed in middle aged female Rhes KO mice, either in the CPu or SNc (Figure 2). Finally, middle aged female Rhes KO mice showed a significant increase of GFAP-positive cells in the CPu compared with the adults (Figure 2).

### CD11b Immunoreactivity

Three-way ANOVA analysis of CD11b immunoreactivity in CPu revealed a significant effect of gender (*F*_(1,44)_ = 122.25, *p* < 0.0001), genotype (*F*_(1,44)_ = 74.57, *p* < 0.0001) and age (*F*_(1,44)_ = 16.56, *p* < 0.0001). Moreover, a significant gender × genotype interaction (*F*_(1,44)_ = 62.65, *p* < 0.0001), gender × age interaction (*F*_(1,44)_ = 24.75, *p* < 0.0001), genotype × age interaction (*F*_(1,44)_ = 52.78, *p* < 0.0001) and gender × genotype × age interaction (*F*_(1,44)_ = 63.75, *p* < 0.0001) were also observed in CPu. Furthermore, three-way ANOVA analysis of CD11b immunoreactivity in SNc revealed significance of gender (*F*_(1,47)_ = 22.90, *p* < 0.0001) and genotype (*F*_(1,47)_ = 30.25, *p* < 0.0001). Finally, a significant gender × genotype interaction (*F*_(1,47)_ = 19.84, *p* < 0.0001), genotype × age interaction (*F*_(1,47)_ = 9.00, *p* < 0.005) and gender × genotype × age interaction (*F*_(1,47)_ = 21.54, *p* < 0.0001) were also observed in SNc.

**Figure 3:**
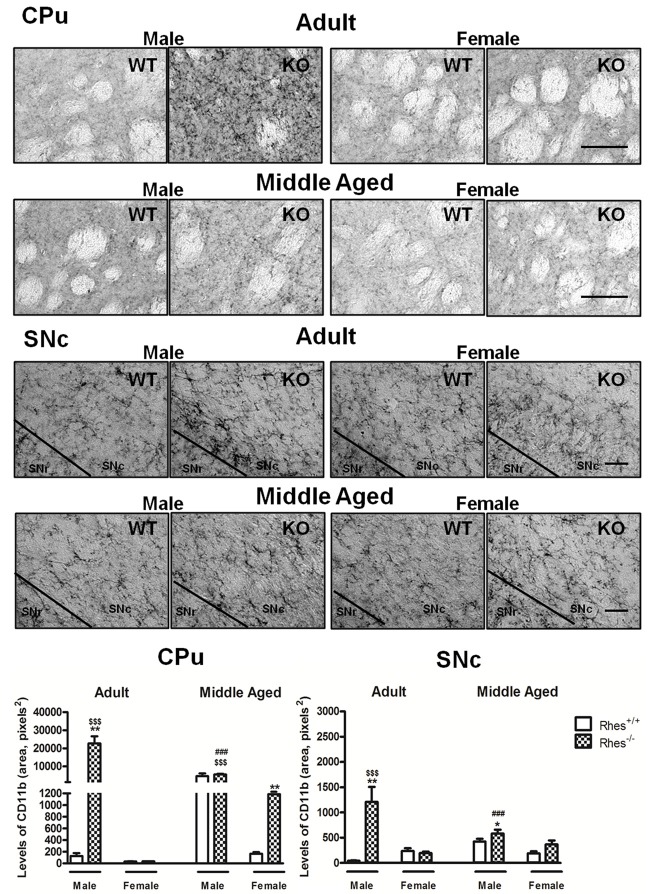
Complement type 3 receptor (CD11b) immunohistochemistry in Rhes WT and Rhes KO mice. Representative sections and histograms of the CPu and SNc immunostained for CD11b of adult and middle aged male and female Rhes WT and Rhes KO mice. The histograms in the left and right panels show the area occupied by gray values above a threshold, calculated and expressed as square pixels in the CPu and SNc, respectively. Values are expressed as mean ± SEM. The number of mice per group is: adult Rhes WT, males *n* = 5 and females *n* = 8; middle aged Rhes WT, males *n* = 12 and females *n* = 6; adult Rhes KO, males *n* = 5 and females *n* = 9; middle aged Rhes KO, males *n* = 8 and females *n* = 6. *p < 0.05, **p < 0.005 compared with Rhes WT mice; ^$$$^*p* < 0.001 compared with female Rhes KO mice; ^###^*p* < 0.001 compared with the respective adult Rhes KO mice by Tukey’s *post hoc* test. Scale bar: 50 μm.

In particular, adult male Rhes KO mice showed a significant increase in the level of CD11b, both in the CPu and SNc, compared with male WT and female Rhes KO mice (Figure 3). No modifications to the levels of CD11b were observed in adult female Rhes KO mice, either in the CPu or SNc (Figure 3).

Middle aged male Rhes KO mice showed a significant increase in the levels of CD11b in the SNc (Figure 3), compared with male WT mice. Middle aged female Rhes KO mice showed a significant increase in CD11b immunostaining in the CPu, but not in the SNc (Figure 3). Finally, middle aged male Rhes KO mice showed a significant increase of CD11b-positive cells in the CPu and SNc compared with the adults (Figure 3).

## Discussion

It was previously shown that lack of Rhes leads to a decrease of nigral TH-positive neurons accompanied by motor coordination deficits in male mice (Pinna et al., 2016). The present study provides insight into the factors contributing to neurodegeneration in Rhes KO mice by demonstrating that, besides the DAergic neurodegeneration, an increase in astrogliosis and microgliosis is present in these KO mice. Moreover, gliosis was age-dependent and more marked in male than in female mice.

The analysis of TH immunoreactivity in the CPu and SNc of Rhes KO mice pointed to a decrease in TH levels, compared with WT mice. Moreover, the evaluation of the relationship between this decrease and gender revealed a further important difference, since male Rhes KO mice showed a more marked decrease than female KO mice. In line with this, in the CPu of adult male Rhes KO mice, a significant decrease in TH-positive fibers was observed compared with WT mice, whereas adult female Rhes KO mice did not display any modification compared with WT mice. The lack of a decrease in TH-positive fibers observed in the CPu of adult female Rhes KO mice may be related to compensatory mechanisms such as sprouting and branching, as it has been described, for example, in experimental PD models and in PD patients (Bezard et al., 2000; Finkelstein et al., 2000; Lee et al., 2008). It is noteworthy that *in vitro* studies demonstrated that estrogens have neurotrophic effects on nigrostriatal DAergic neurons, promoting the growth of neurites that expresses TH (Reisert et al., 1987; Beyer and Karolczak, 2000). Furthermore, other *in vitro* studies revealed that estrogens enhanced the differentiation and survival of DAergic neurons derived from human neuronal stem cells (Kishi et al., 2005). These studies were sustained by later studies in female mice, where estrogens administration increased the nigrostriatal TH expression (Ivanova and Beyer, 2003). Collectively, these data are in line with clinical studies reported in the introduction, where a lower incidence of PD was found in women as compared to men, and results obtained in Rhes KO mice seems to confirm this difference. However, middle aged male Rhes KO mice showed a higher number of TH-positive neurons in the SNc compared with female mice. It is feasible to hypothesize that during aging in pathological conditions, such as neurological disorders, the relative resistance to the damage of female may be lost, or at least compromised in the SNc, but not in the CPu. Collectively, these results, by showing that DA nigrostriatal neurons of female Rhes KO mice have a lower vulnerability in the CPu with respect to male Rhes KO mice, added further interest to this animal model.

Although only further studies will clarify the general mechanism at the basis of DA neuron degeneration in male and female Rhes KO mice, it is interesting to evidence that previous *in vitro* and *in vivo* studies found that Rhes mRNA is localized in neurons rather than glial cells (Vargiu et al., 2004; Errico et al., 2008; Ghiglieri et al., 2015; Sciamanna et al., 2015; Vitucci et al., 2015).

Regarding the mechanisms at the basis of the present results, we may hypothesized, on the basis of reports listed below, the involvement of several signal pathways implicated in neuronal survival, such as the mammalian target of rapamycin (mTOR), a serine–threonine kinase that coordinates cell growth in response to environmental cues (Laplante and Sabatini, 2012), and Beclin, a protein able to interact with either Bcl-2 or phosphoinositide 3-kinase class III and regulate both autophagy and cell death (Mealer et al., 2014). Moreover, the overexpression of Rhes in cells, results in marked autophagic flux activation (Mealer et al., 2014). This autophagic enhancement mediated by Rhes reflects its ability to isolate Beclin-1 and avoid the binding of Bcl-2 (Mealer et al., 2014). Furthermore, *in vitro* and *in vivo* evidence has revealed that the calcium channel dysregulation may be linked to a number of disorders, including PD; in this regard, it has been found that Rhes influences a specific type of calcium channel, the N-type/Cav2.2, reducing the basal calcium current density and thereby influencing calcium-dependent events, such as α-synuclein aggregation (Thapliyal et al., 2008; Sciamanna et al., 2015). Finally, since Rhes plays a crucial role in iron homeostasis (Choi et al., 2013), that represents one of the causes of neuron vulnerability, the perturbation in iron homeostasis in Rhes KO mice might contribute to a disruption of DAergic function.

As reported in the “Introduction” section, emerging evidence indicates that neuroinflammation may play a role in the nigral DA neuron degeneration observed in PD (Barcia et al., 2009; Tansey and Goldberg, 2010; Halliday and Stevens, 2011; Hirsch et al., 2012; Phani et al., 2012). In line with this, degenerating DAergic neurons in the SNc of PD patients are paired with microglia activation and accumulation of proinflammatory factors (McGeer et al., 1988; Teismann and Schulz, 2004; Lull and Block, 2010), as well as in various PD animal models (Gao et al., 2003; Costa et al., 2013, 2014). The analysis of GFAP immunoreactivity in the CPu and SNc of Rhes KO mice revealed, in general, a different result between male and female mice. While adult male Rhes KO mice displayed astrogliosis in the CPu, female Rhes KO mice did not display this activation. Moreover, with aging, male Rhes KO mice displayed significant astrogliosis in the SNc, whereas female KO mice were resistant, even when middle aged. This difference between male and female Rhes KO mice in GFAP levels did not surprise, since it was previously reported that astrocytes may participate in the generation of gender differences in the etiology of neurological disorders or in the response of the brain to pathological insults (Santos-Galindo et al., 2011).

As already observed for GFAP immunoreactivity, the analysis of CD11b immunoreactivity in the CPu and SNc of male Rhes KO mice showed, in general, a more marked increase in the levels of CD11b compared with female mice. In this respect, adult and middle aged male Rhes KO mice showed high levels of CD11b immunoreactivity in both the CPu and SNc compared with female KO mice. These results are associated in female Rhes KO mice with a relative resistance of microgliosis in the CPu and SNc in adult mice and in the SNc in middle aged mice. As for GFAP, these results could be explained in light of previous *in vitro* studies that have shown how estrogens are able to attenuate microglial superoxide release and phagocytic activity (Bruce-Keller et al., 2000) and to block the phenotypic conversion associated with microglial activation, preventing the production of inflammatory mediators (Vegeto et al., 2001). Ongoing studies will clarify which of these proposed mechanisms are involved.

Although in the last decade several chemical animal models of PD have been established, most of them missed some pathophysiological features of the disease (Potashkin et al., 2010; Bezard et al., 2013; Folch et al., 2017). Moreover, in contrast to the experimental models of DA neuron degeneration based on toxic insults, in which a toxin is injected locally in different basal ganglia structures, the Rhes KO animal model might provide new insights into the basal ganglia structures more vulnerable to DA neuron degeneration and might help in clarifying the mechanism of idiopathic PD, where the mechanism of neurodegeneration of DA neurons is still unclear. Furthermore, most of the toxin-based or genetic PD models do not point out the differential gender incidence of the pathology in humans, using, in general, male animals without the comparison with the respective females (Potashkin et al., 2010).

In conclusion, as suggested in our previous article (Pinna et al., 2016), Rhes KO mice might represent a genetic model of a mild PD-like phenotype and the results reported in the present study may give further support to Rhes KO mice as a suitable model to study mechanisms and vulnerability factors of DAergic neurons degeneration as in PD.

## Author Contributions

GC and PFP performed the immunostaining experiments. GC and MAC analyzed data. FN and ADM bred the mice colony. GC, AP and MM wrote the manuscript. GC, AP, AU and MM reviewed and edited the manuscript. All authors read and approved the final manuscript.

## Conflict of Interest Statement

The authors declare that the research was conducted in the absence of any commercial or financial relationships that could be construed as a potential conflict of interest.

## Abbreviations

ABC: avidin–biotin–peroxidase complex
CD11b: complement type 3 receptor
CPu: caudate putamen
DA: dopamine
DAergic: dopaminergic
GFAP: glial fibrillary acidic protein
KO: knockout
mTOR: mammalian target of rapamycin
PD: Parkinson’s disease
SNc: substantia nigra pars compacta
TH: tyrosine hydroxylase
WT: wild-type.
