# Supplementary material for: Rhes Counteracts Dopamine Neuron Degeneration and Neuroinflammation Depending on Gender and Age
Source: Front Aging Neurosci. 2018 May 31;10:163. doi: 10.3389/fnagi.2018.00163 (PMC5990628; doi:10.3389/fnagi.2018.00163)
Supplement: Supplementary file 1 [file Table_1.DOCX]

**Rhes counteracts dopamine neuron degeneration and neuroinflammation depending on gender and age**

Giulia Costa^1†^, Annalisa Pinna^2†^*, Pier Francesca Porceddu^1^, Maria Antonietta Casu^4^, Anna Di Maio^5^, Francesco Napolitano^6,8^, Alessandro Usiello^6,7^, Micaela Morelli^1,2,3^

^1^Department of Biomedical Sciences, Section of Neuropsychopharmacology, University of Cagliari, Cagliari, Italy

^2^National Research Council of Italy, Neuroscience Institute, Cagliari, Italy

^3^National Institute of Neuroscience (INN), University of Cagliari, Cagliari, Italy

^4^National Research Council of Italy, Institute of Translational Pharmacology, UOS of Cagliari, Scientific and Technological Park of Sardinia POLARIS, Pula, Italy

^5^IRCCS Casa Sollievo della Sofferenza, 71013 San Giovanni Rotondo, Foggia, Italy

^6^Laboratory of Behavioral Neuroscience, Ceinge Biotecnologie Avanzate, 80145 Naples, Italy.

^7^Department of Environmental, Biological and Pharmaceutical Sciences and Technologies, University of Campania, Luigi Vanvitelli, Caserta, Italy.

^8^Department of Molecular Medicine and Medical Biotechnology, University of Naples "Federico II", 80131 Naples, Italy

^†^These authors equally contributed to the work.

Address correspondence to Annalisa Pinna*, National Research Council of Italy (CNR), Neuroscience Institute - Cagliari, Cittadella Universitaria, Blocco A, 09042 Monserrato (CA), Italy. e-mail: apinna@unica.it; apinna@in.cnr.it

**Running title**: Rhes influences age/gender-dependent nigrostriatal modifications.

|  | Male Rhes WT mice | Male Rhes KO mice | Female Rhes WT mice | Female Rhes KO mice |
| --- | --- | --- | --- | --- |
| **Adult** | 16083 ± 326.4 | 9044 ± 115 | 25021 ± 96 | 21117 ± 476 |
| **Middle Aged** | 17118 ± 86 | 13015 ± 52 | 17058 ± 36 | 10487 ± 31 |

**Table 1.** Results of Nissl cell count in the SNc. Values are expressed as mean ± SEM. The number of mice per group is: adult Rhes WT, males n=5 and females n=8; middle aged Rhes WT, males n=12 and females n=6; adult Rhes KO, males n=5 and females n=9; middle aged Rhes KO, males n=8 and females n=6.
